# Supplementary material for: Collaborative intelligence in AI: Evaluating the performance of a council of AIs on the USMLE
Source: PLOS Digit Health. 2025 Oct 9;4(10):e0000787. doi: 10.1371/journal.pdig.0000787 (PMC12510544; doi:10.1371/journal.pdig.0000787)
Supplement: S2 File — (ZIP) [file pdig.0000787.s002.zip › S2 Data Raw data files containing unprocessed question inputs and LLM outputs including transcripts of deliberations/STEP3_Question-11-1_Type-MC-NJ.docx]

**Facilitator** **[Round 1]:** A 57-year-old woman comes to the office for a preoperative evaluation 2 weeks before undergoing scheduled laparoscopic cholecystectomy. Medical history is otherwise unremarkable and the patient takes no medications. Family history is significant for stable angina in her father and rheumatoid arthritis in her mother. The patient has a 102-year-old grandmother who resides in a nursing care facility and has Parkinson disease. The patient does not smoke cigarettes or drink alcoholic beverages. During the interview, her face is expressionless. She has a flexed posture and is unable to open her mouth wide. She is 173 cm (5 ft 8 in) tall and weighs 81 kg (179 lb); BMI is 27 kg/m2. Vital signs are normal. Physical examination discloses thickening and hardening of the skin over the dorsum of the hands and forearms, as well as mild kyphosis. Strength testing shows no abnormalities; muscle tension is normal. Passive and active range of motion of the upper extremities is full. Gait is slow and deliberate. The remainder of the physical examination discloses no abnormalities. Prior to surgery, further evaluation is indicated for which of the following conditions in this patient? (A) Osteitis deformans (Paget disease) (B) Parkinson disease (C) Progressive supranuclear palsy (D) Sarcopenia (E) Systemic sclerosis (scleroderma)

**AI_E:** RESTATING THE QUESTION: The problem is that a 57-year-old woman who will be having a laparoscopic cholecystectomy in the near future exhibits certain signs, including a lack of facial expression, a flexed posture, limited mouth opening, skin thickening and hardening on the backside of her hands and forearms, mild kyphosis, and a slow, deliberate gait. The task is to determine what condition she should be evaluated further for. The options are osteitis deformans (Paget disease), Parkinson's disease, progressive supranuclear palsy, sarcopenia, and systemic sclerosis (scleroderma). PLANNING: The assumptions here are that the signs demonstrated by the patient are indicative of a specific condition and that one of the options provided is the correct choice. We could question whether there might be other conditions at play or whether various signs could indicate different conditions without one overarching condition accounting for all of them. Given the presence of several characteristic signs, such as skin changes, slow gait, flexed posture and difficulty in opening mouth wide, it is reasonable to hypothesize that the patient may be suffering from a systemic disease such as scleroderma (systemic sclerosis). CLINICAL REASONING: The slow gait and expressionless face might remind one of Parkinson's disease, but the patient doesn't display the characteristic resting tremors of Parkinson's. Systemic sclerosis (scleroderma) often results in a tight face (which could explain her expressionless face and difficulty in opening mouth wide) and could explain the hardening and thickening of skin over hands and forearms. Scleroderma would also integrate the kyphosis, as part of the systemic connective tissue involvement of scleroderma could lead to some skeletal deformities. CONCLUDING: Considering the overall clinical presentation and the characteristic physical findings, it seems rational to suggest further evaluation for the systemic sclerosis (scleroderma). RESPONSE: Systemic sclerosis (scleroderma) (Choice E) should be evaluated further in this patient.

**AI_A:** RESTATING THE QUESTION: A 57-year-old woman is scheduled to undergo a laparoscopic cholecystectomy, a minimally invasive surgical procedure to remove the gallbladder. The patient has a few clinical signs such as an expressionless face, a flexed posture, and limitations in opening her mouth wide. Physical examination findings include hardened and thickened skin over her hands and forearms and a slightly hunched back (mild kyphosis). Her gait is slow and deliberate. The question is about which condition should be evaluated further in this patient before proceeding with the scheduled surgery. PLANNING: The assumptions include considering the patient's clinical presentation, family history, and physical examination findings. The family history suggests a genetic predisposition to neurological disorders (Parkinson disease in her grandmother). The patient's expressionless face, flexed posture, inability to open her mouth wide, and slow and deliberate walk suggest a possible neurological condition like Parkinson's disease. The findings of thickened and hardened skin may suggest a connective tissue disorder like systemic sclerosis. However, each assumption must be questioned. For example, the hardened skin may be related to a skin disease rather than a systemic disorder. Similarly, her posture and face could be due to a musculoskeletal condition rather than a neurological disease. Given the constellation of symptoms, Parkinson's disease seems a clinically reasonable priority which the patient requires further evaluation for before the surgery. CLINICAL REASONING: Parkinson's disease, a neurodegenerative disorder, is characterized early on by a reduction in facial expressions (hypomimia), flexed posture, slower volitional movements (bradykinesia), and masqueraders like the inability to open the mouth wide due to rigidity. These symptoms corroborate with the patient's presentation. Although the skin changes could be explained by conditions like systemic sclerosis, the lack of associated symptoms such as systemic manifestations (like Raynaud's phenomenon, dysphagia, or tight skin over face) and the absence of specific organ disease (like interstitial lung disease, pulmonary hypertension) make this less likely. CONCLUDING: Considering her clinical presentation, family history, and physical examination findings, it's reasonable to be concerned about the patient's potential neurological condition. Given that she has elements of Parkinsonism and a family history of Parkinson's disease, option B (Parkinson's disease) would be clinically prudent. RESPONSE: Prior to her surgery, this patient requires further evaluation for Parkinson disease (B). This neurodegenerative disorder is a possibility due to her symptom profile and family history. Importantly, an untreated or advanced case might complicate her surgery and post-operative recovery. As such, a comprehensive neurological evaluation should be done to establish a diagnosis and develop an appropriate care plan before her operation.

**AI_D:** RESTATING THE QUESTION: The patient to be evaluated is a 57-year-old female who is scheduled for a laparoscopic cholecystectomy in two weeks. She has an unremarkable medical history and is not taking any medications. She does not drink or smoke cigarettes. A couple of diseases such as rheumatoid arthritis and stable angina run in her family, and her grandmother, aged 102, who lives in a nursing home, has Parkinson's disease. During her physical examination, the patient's face is inexpressive, her posture is flexed and she seems unable to open her mouth widely. She measure 5'8" tall, with a weight of 179lbs, resulting in a BMI of 27 kg/m². The examination also revealed hardening and thickening of the hand and forearm skin as well as a slight kyphosis. Her upper extremities have full range of motion, both active and passive, and her muscle tone and strength seem normal. She walks slowly and purposefully. PLANNING: The underlying assumptions here are: 1) This patient may have Parkinson's disease given her family history and the clinical findings. 2) The patient may be experiencing symptoms of a systemic disease such as Systemic sclerosis (scleroderma) given her physical manifestation of thickened and hardened skin. 3) Other possible diagnoses like Osteitis deformans (Paget's disease), Progressive Supranuclear Palsy, and Sarcopenia should be considered. CLINICAL REASONING: Although her family history suggests that her grandmother has Parkinson's disease, the patient's symptoms are not entirely typical of it due to the absence of resting tremors, bradykinesia, rigidity, and postural instability. Moreover, symptoms of Parkinson's disease do not usually include thickening and hardening of the skin on the hands and forearms. Therefore, assumption 1 may not be the most probable. Assumption 2, systemic sclerosis (scleroderma) should be prioritized for further evaluation due to the presence of hallmark signs of sclerosis - thickening and hardening of the skin on her hands and forearms. Scleroderma would also typically present with symptoms such as a tight face and limited ability to open the mouth wide - which she also exhibits. Other choices in the question do not fit the patient's presentation. So, the possibility of Progressive Supranuclear Palsy, Sarcopenia, and Osteitis Deformans (Paget's disease) are less likely. CONCLUDING: In conclusion, this patient is exhibiting symptoms consistent with Systemic sclerosis (scleroderma). Her skin manifestations, along with her limited ability to open her mouth wide, point towards a possible diagnosis of scleroderma, a connective tissue disease characterized by fibrosis of the skin and internal organs. RESPONSE: Therefore, further evaluation for Systemic Sclerosis (Scleroderma) (Option E) is indicated in this patient prior to her scheduled surgery.

**AI_C:** RESTATING THE QUESTION: A 57-year-old woman, planning a laparoscopic cholecystectomy, presents with an expressionless face, a limited ability to open her mouth wide, a flexed posture, thickening and hardening of the skin on the upper extremities, mild kyphosis, and a slow, deliberate gait. Her family has a history of stable angina, rheumatoid arthritis and Parkinson's disease. The patient is otherwise healthy in her medical and social history. She is now seeking a preoperative evaluation and the question asks which condition needs to be evaluated further in this patient. The conditions listed are: Osteitis deformans (Paget disease), Parkinson disease, Progressive supranuclear palsy, Sarcopenia, and Systemic sclerosis (scleroderma). PLANNING: The patient has some symptoms that overlap with multiple conditions listed. For instance, the thickening of skin and limited mouth-opening ability could suggest systemic sclerosis, while her slow, deliberate gait and expressionless face may indicate Parkinson's disease. Yet, the absence of tremors and a relevant family history makes it prudent to explore other possibilities too. Having ruled out Paget's disease (due to the absence of a history of bone pain) and Progressive supranuclear palsy (which could cause unsteady gait but would feature vertical gaze palsy not seen in this patient), and sarcopenia (as the patient's muscle tension and strength appear normal), the intriguing alternate assumption is systemic sclerosis which could explain mouth and skin symptoms, and potentially link to her slow, deliberate gait. CLINICAL REASONING: Let's consider systemic sclerosis (scleroderma) closer. This is a chronic connective tissue disease of unknown etiology that often presents in patients in their 30s-50s, and is more common in women - fitting our patient's demographic. A key trademark of scleroderma is the thickening and hardening of the skin due to excess collagen produced by fibroblasts. This is consistent with what is seen on the physical examination of the patient - thickening and hardening of the skin over the hands and forearms. Scleroderma could also explain the patient's inability to open her mouth wide - the skin hardening over time can, in severe cases, restrict mouth movement. Moreover, microvascular dysfunction and fibrosis may potentially contribute to her slow, deliberate gait. CONCLUDING: In conclusion, although the patient has a family history of Parkinson's her other symptoms - restricted mouth opening, thickened skin on hands and forearms - hint more towards systemic sclerosis (scleroderma). The evaluation of her gait, muscle strength, and other physical tests do not hint at more obvious neurodegenerative diseases, leaving scleroderma as a higher priority for further examination for this individual. RESPONSE: Therefore, prior to surgery, further evaluation is indicated for Systemic sclerosis (scleroderma) (Option E) in this patient.

**AI_B:** Restating the Question: A healthy 57-year-old woman is awaiting a laparoscopic cholecystectomy and undergoes a preoperative evaluation. She has no history of chronic illness and is not on any medication. She has a family history of cardiovascular disease and rheumatoid arthritis. Additionally, she has a grandmother, who at the age of 102 resides in a nursing care facility and has Parkinson's disease. Despite no history of smoking or drinking, the patient presents with an expressionless face, flexed posture, and difficulty in fully opening her mouth. Her physical attributes include a height of 173 cm, a weight of 81 kg and a BMI of 27 kg/m2. Her skin is observed to be unusually thick and hard, specifically over the dorsum of the hands and forearms, and she has a mild hunchback (kyphosis). Muscle strength and tone are normal, and she has a full range of motion of upper limbs. However, her walk is slow and she appears to be very deliberate with her movements. With all these considerations, the query is to identify which condition needs further evaluation prior to her surgery. Planning The woman presents non-specific symptoms like an expressionless face, difficulty opening her mouth widely, flexed posture, slow and deliberate gait, combined with specific symptoms of skin thickening and hardening over the hands and forearms, as well as mild kyphosis. These could suggest conditions like (A) Osteitis deformans (B) Parkinson's disease (C) Progressive supranuclear palsy (D) Sarcopenia or (E) Systemic sclerosis (scleroderma). But, amongst these, consideration of Systemic sclerosis is prioritized due to the manifestation of skin-related symptoms, which is not seen in the other conditions. Clinical Reasoning: Systemic sclerosis (scleroderma) is a connective tissue disorder that affects multiple systems, but most notably causes skin thickening. The facial expressionlessness, difficulty opening the mouth (microstomia), skin thickening over the hand and forearm, and flexed posture are consistent with this condition. Though the patient's slow, deliberate gait, expressionless face, and flexed posture might suggest Parkinson's disease (given her family history), the lack of tremors or other motor abnormalities drives the probability towards scleroderma. Concluding: Upon reviewing the patient's symptoms and the indicative conditions, the possibility of systemic sclerosis seems to align best with the patient's presentation, as it provides a connectivity between the skin abnormalities and other manifestations, like expressionless face and difficulty to open the mouth widely. Thus, before proceeding with her surgery, it would be prudent to evaluate for systemic sclerosis in this patient. Response: The patient's features, such as the thickening and hardening of the skin, minimal facial expressions, and difficulty in opening her mouth wide, are suggestive of Systemic sclerosis (scleroderma) (E). Hence, she should be further evaluated for this condition.

**Facilitator** **[Round 2]:** Summary: AI_A believes that the patient's symptoms suggest a neurological condition like Parkinson's disease, given her family history and the presence of symptoms such as an expressionless face, flexed posture, and slow, deliberate walk. AI_A suggests that the patient requires further evaluation for Parkinson's disease before the surgery. AI_B, AI_C, AI_D, and AI_E all suggest that the patient's symptoms, including skin thickening and hardening over the hands and forearms, minimal facial expressions, and difficulty in opening her mouth wide, are indicative of Systemic sclerosis (scleroderma). They all recommend that the patient should be further evaluated for this condition. Question to clarify disagreements: The main disagreement between the council members' opinions lies in the interpretation of the patient's symptoms and their relation to either Parkinson's disease or Systemic sclerosis (scleroderma). To clarify this disagreement, the following question could be asked: "What specific symptoms or combination of symptoms led you to your conclusion, and how do you justify prioritizing these symptoms over others in diagnosing either Parkinson's disease or Systemic sclerosis (scleroderma)?". Based on your preceding response, how would you answer the original question: [A 57-year-old woman comes to the office for a preoperative evaluation 2 weeks before undergoing scheduled laparoscopic cholecystectomy. Medical history is otherwise unremarkable and the patient takes no medications. Family history is significant for stable angina in her father and rheumatoid arthritis in her mother. The patient has a 102-year-old grandmother who resides in a nursing care facility and has Parkinson disease. The patient does not smoke cigarettes or drink alcoholic beverages. During the interview, her face is expressionless. She has a flexed posture and is unable to open her mouth wide. She is 173 cm (5 ft 8 in) tall and weighs 81 kg (179 lb); BMI is 27 kg/m2. Vital signs are normal. Physical examination discloses thickening and hardening of the skin over the dorsum of the hands and forearms, as well as mild kyphosis. Strength testing shows no abnormalities; muscle tension is normal. Passive and active range of motion of the upper extremities is full. Gait is slow and deliberate. The remainder of the physical examination discloses no abnormalities. Prior to surgery, further evaluation is indicated for which of the following conditions in this patient? (A) Osteitis deformans (Paget disease) (B) Parkinson disease (C) Progressive supranuclear palsy (D) Sarcopenia (E) Systemic sclerosis (scleroderma)]

**AI_E:** The patient's symptoms present some degree of complexity as they seem to span across two different categories, namely neurological and rheumatological conditions. While her history of flexed posture, expressionless face, slow gait could point towards Parkinson's disease, her skin manifestations, inability to open her mouth wide are suggestive of Systemic sclerosis (scleroderma) - a connective tissue disorder. However, as an AI with a thorough understanding of medical knowledge, I would prioritize the existence of both skin and musculoskeletal symptoms over the neurological ones. Considering the patient's thickening skin and the phenomenon of "mask face" (inability to fully open the mouth) are among the classic symptoms of scleroderma, it makes this condition more probable in light of the patient's symptoms. Furthermore, while flexed posture can indeed be observed in Parkinson's disease, it's also seen in scleroderma due to joint rigidity. The existence of mild kyphosis further backs up this claim as it could be a result of epiphenomena of scleroderma like interstitial lung disease. The patient's slow, deliberate gait could also be attributed to musculoskeletal discomfort due to the same condition. Given the wider constellation of the symptoms in the context of Systemic sclerosis (scleroderma), it seems reasonable to pursue an evaluation for this condition in this patient. However, this doesn't necessarily rule out co-occurrence of Parkinson's disease, and a separate evaluation could be considered once the scleroderma diagnosis or treatment plan have been formalized. In conclusion, based the on above clinical reasoning, the patient should be further evaluated for Systemic sclerosis (scleroderma) (E) before the surgery. It is notable that the management of scleroderma might influence the surgical procedure, anesthesia, and post-operation care.

**AI_A:** RESTATING THE QUESTION: The clinical scenario depicted explores a 57-year-old woman presenting for a preoperative evaluation prior to a scheduled laparoscopic cholecystectomy. She exhibits several symptoms, such as an expressionless face, flexed posture, inability to open her mouth wide, thickening and hardening of the skin over the hands and forearms, and a slow and deliberate gait. The question asks for the recommendation of further evaluation of a certain condition with multiple choices provided. PLANNING: Notably, her symptoms include thickened skin on her hands and forearms, a slack face, and difficulty opening her mouth wide. Also important is her slow, deliberate gait. The aforementioned symptoms are characteristic of certain systemic or neurological diseases. Her family history includes Parkinson's disease, but it's critical to consider the likelihood of each condition independently. CLINICAL REASONING: Each selected choice should be considered in light of her presentation. (A) Osteitis deformans (Paget disease) mainly affect the bones and wouldn't account for the patient's facial condition or skin changes, (B) Parkinson's Disease could explain her facial expression and gait but would not account for the skin changes, (C) Progressive supranuclear palsy is a possible explanation for her gait and facial features, but it also doesn't account for the skin symptoms, (D) Sarcopenia, or age-related loss of muscle mass, might explain her posture but doesn't account for her facial or skin condition, (E) Systemic sclerosis (scleroderma), an autoimmune connective tissue disease, often presents with skin thickening and contractures. It can also affect the muscles and lead to facial and postural changes. CONCLUDING: Given the patient's presentation and associated symptoms, her condition seems more likely to be Systemic Sclerosis (scleroderma) which affects the skin and muscles rather than a neurological condition like Parkinson's or an age-related loss of muscle mass like sarcopenia. RESPONSE: The patient requires further evaluation for Systemic sclerosis (scleroderma) (E).

**AI_C:** 1) RESTATING THE QUESTION: The question is asking which medical condition should be further evaluated in a 57-year-old woman before she undergoes a scheduled surgical procedure, based on a set of physical and medical observations laid out in the prompt. 2) PLANNING: Two main assumptions stand out from the prompt: The patient may be suffering from Parkinson's disease based on her facial expression, gait, and family history. On the other hand, the patient might also be suffering from Systemic sclerosis (scleroderma) because of the appearance of her skin. Questioning these assumptions leads to two possible scenarios: one prioritizing the neurological signs and the other considering the dermatological symptoms. Clinically, it is essential to evaluate which condition could have greater repercussions on the patient's surgery and overall health status. 3) CLINICAL REASONING: The assumption of Parkinson's disease is supported primarily by the patient's gait, expressionless face, and family history. However, while these are definite signs of Parkinson's, they should often be accompanied by other symptoms such as tremors, which are not mentioned here. Additionally, Parkinson’s disease in her grandmother may not increase her risk, considering Parkinson's is typically sporadic and not familial. On the other hand, scleroderma can present with symptoms including hardening and thickening of the skin which she has, plus it could cause an expressionless face and difficulty opening her mouth due to skin thickening. Scleroderma can lead to complications such as gastrointestinal bleeding or perforation during surgery due to affected GI motility, hence evaluation is warranted prior to surgery. 4) CONCLUDING: Looking at every symptom in context and prioritizing those that could affect the preoperative evaluation for the scheduled surgery, I lean towards the assumption that systemic sclerosis (scleroderma) could be more relevant in this case. While the neurological signs remain important, they do not seem to present an immediate risk to her upcoming surgical procedure, but scleroderma could. 5) RESPONSE: Considering all clinical signs and their implications, I believe further evaluation is indicated for systemic sclerosis (scleroderma) (Option E) in this patient before undergoing her scheduled laparoscopic cholecystectomy.

**AI_D:** Restating the Question: The query seeks to determine the medical condition that should warrant further evaluation in a 57-year-old woman who is currently undergoing preoperative assessment for a laparoscopic cholecystectomy. She has an unremarkable medical history but her family history includes stable angina in her father and rheumatoid arthritis in her mother. She also has a grandmother with Parkinson's disease. The patient demonstrates an expressionless face, inability to open her mouth wide, slow and deliberate gait, and a flexed posture. Physical examination reveals hardening and thickening of the skin on her hands and forearms, normal muscle strength and tension, and full range of motion in her upper extremities. Planning: To resolve the query, we first need to consider the central symptoms: the skin hardening and thickening, facial expressional changes, and the limitations in mouth opening. We can hypothesize that these indicate a connective tissue disorder or a neurological pathology. Because there's a family history of Parkinson's disease, this could guide our hypothesis toward a similar neurological condition. However, while the Parkinson's disease could be misleading us, systemic sclerosis (scleroderma) could also be a viable explanation due to the skin and facial changes observed. Clinical Reasoning: Scleroderma would explain many symptoms, but it does not usually lead to an expressionless face and slow gait. On the other hand, while Parkinson's disease would account for these two symptoms, and there's a family history, the skin symptoms are atypical for Parkinson's. Therefore, since the majority of symptoms lean towards scleroderma, it should be prioritized. It is common clinical practice to rule out diseases that could result in severe consequences if left undiagnosed, and scleroderma falls into this category. Concluding: The skin hardness and thickening in conjunction with her facial changes are suggestive of scleroderma, a systemic disorder. Her slow and deliberate gait and flexed posture also align with movement impairments seen in scleroderma. This condition, if left undiagnosed and untreated, can have more severe implications, hence, should be diagnosed promptly. Response: From the combination of symptoms, family history, and clinical implications of potential disorders, it can be inferred that this patient requires further investigation for systemic sclerosis (scleroderma), therefore, the answer is (E) Systemic sclerosis (scleroderma).

**AI_B:** RESTATING THE QUESTION: The given clinical situation is about a 57-year-old woman scheduled to undergo a laparoscopic cholecystectomy in a fortnight. Her relevant clinical findings include an expressionless face, difficulties in opening her mouth wide, thickening and hardening of the skin over the hands and forearms, a stooped posture, and a slow, deliberate gait. The primary question is to determine the condition for which this patient should be further evaluated prior to surgery. PLANNING: The priority here is to analyze her symptoms and her family history to determine the underlying condition, which is either neurological (Parkinson's disease, Progressive supranuclear palsy) or a systemic disease such as Systemic sclerosis (scleroderma) or Osteitis deformans (Paget disease). It is also important to consider the generically aging-associated condition like Sarcopenia. CLINICAL REASONING: Her symptoms such as facial expressionlessness, deliberate gait, and mild kyphosis might suggest Parkinson's disease, especially given her 102-year-old grandmother's history of Parkinson's. However, the robustness of our symptoms directed towards neurodegenerative diseases is not present, such as tremor, rigidity, or bradykinesia. In contrast, the patient's significant symptom is the hardening and thickening of her skin over the hands and forearms, along with a subtle inability to open her mouth wide, which could direct us towards a connective tissue disorder, specifically, Systemic sclerosis (scleroderma). CONCLUDING: Given that Systemic sclerosis (scleroderma) also accounts for the non-specific features of this patient like stooped posture and slower gait, it's reasonable to prioritize this condition for further evaluation, especially since timely intervention can prevent further complications related to the disease like lung, heart, and kidney problems - which could be important for a patient about to undergo surgery. RESPONSE: In conclusion, considering all the given clinical presentations and their prevalences, I believe this patient should be further evaluated for Systemic sclerosis (scleroderma) - choice (E) before proceeding with her surgery.

**Facilitator** The consensus among the AI responses is that the 57-year-old woman, who is scheduled for a laparoscopic cholecystectomy, should be further evaluated for Systemic sclerosis (scleroderma) before proceeding with her surgery. This conclusion is based on her symptoms, which include an expressionless face, inability to open her mouth wide, thickening and hardening of the skin over the hands and forearms, a flexed posture, and a slow, deliberate gait. While some of these symptoms could suggest a neurological condition like Parkinson's disease, especially considering her family history, the majority of her symptoms, particularly the skin changes, are more indicative of a connective tissue disorder like Systemic sclerosis (scleroderma). This condition could have significant implications for her surgery and overall health status, making it crucial to evaluate and manage it promptly. Therefore, the consensus is that the patient should be further evaluated for Systemic sclerosis (scleroderma) (Option E).
